# Should we use closed or open infusion containers for prevention of bloodstream infections?

**DOI:** 10.1186/1476-0711-9-6

**Published:** 2010-02-02

**Authors:** Manuel S Rangel-Frausto, Francisco Higuera-Ramirez, Jose Martinez-Soto, Victor D Rosenthal

**Affiliations:** 1 Century XXI Specialties IMSS Hospital, Mexico City, Mexico; 2 General Hospital, Mexico City, Mexico; 3 Gabriel Mancera IMSS Hospital, Mexico City, Mexico; 4 Medical College of Buenos Aires, Argentina

## Abstract

**Background:**

Hospitalized patients in critical care settings are at risk for bloodstream infections (BSI). Most BSIs originate from a central line (CL), and they increase length of stay, cost, and mortality. Open infusion containers may increase the risk of contamination and administration-related (CLAB) because they allow the entry of air into the system, thereby also providing an opportunity for microbial entry. Closed infusion containers were designed to overcome this flaw. However, open infusion containers are still widely used throughout the world.

The objective of the study was to determine the effect of switching from open (glass, burettes, and semi-rigid) infusion containers to closed, fully collapsible, plastic infusion containers (Viaflex^®^) on the rate and time to onset of central line-associated bloodstream infections CLABs.

**Methods:**

An open label, prospective cohort, active healthcare-associated infection surveillance, sequential study was conducted in four ICUs in Mexico. Centers for Disease Control National Nosocomial Infections Surveillance Systems definitions were used to define device-associated infections.

**Results:**

A total of 1,096 adult patients who had a central line in place for >24 hours were enrolled. The CLAB rate was significantly higher during the open versus the closed container period (16.1 versus 3.2 CLAB/1000 central line days; RR = 0.20, 95% CI = 0.11-0.36, P < 0.0001). The probability of developing CLAB remained relatively constant in the closed container period (1.4% Days 2-4 to 0.5% Days 8-10), but increased in the open container period (4.9% Days 2-4 to 5.4% Days 8-10). The chance of acquiring a CLAB was significantly decreased (81%) in the closed container period (Cox proportional hazard ratio 0.19, P < 0.0001). Mortality was statistically significantly lower during the closed versus the open container period (23.4% versus 16.1%; RR = 0.69, 95% CI = 0.54-0.88, P < 0.01).

**Conclusions:**

Closed infusion containers significantly reduced CLAB rate, the probability of acquiring CLAB, and mortality.

## Background

Patients in hospitals are at risk for bloodstream infections (BSI), mainly in critical care settings. Most BSIs originate from a central line (CL) [1], and they increase length of stay, cost, and mortality [2-14].

During setup, admixture preparation, and administration [15,16], there is a high risk of contamination of intravenous (IV) fluids. When the system is vented, as is mandatory with open infusion containers, there are extra risks of extrinsic contamination.

**Figure 1:**
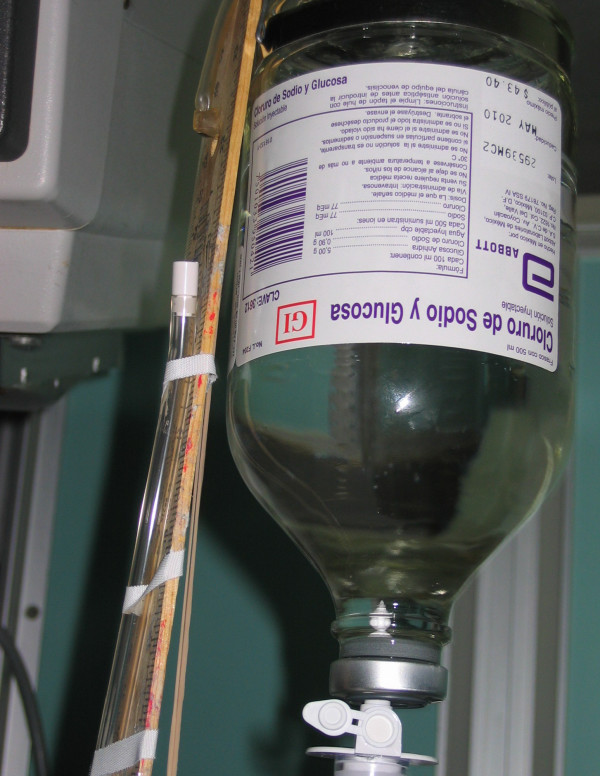
**Open Infusion Container - Glass container with air filter**.

**Figure 2:**
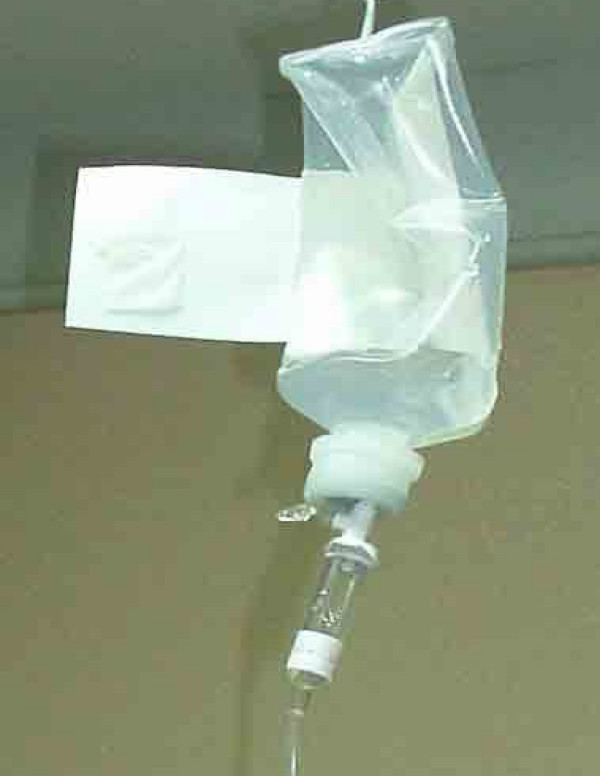
**Open Infusion Container - Semi-rigid container with air filter**.

**Figure 3:**
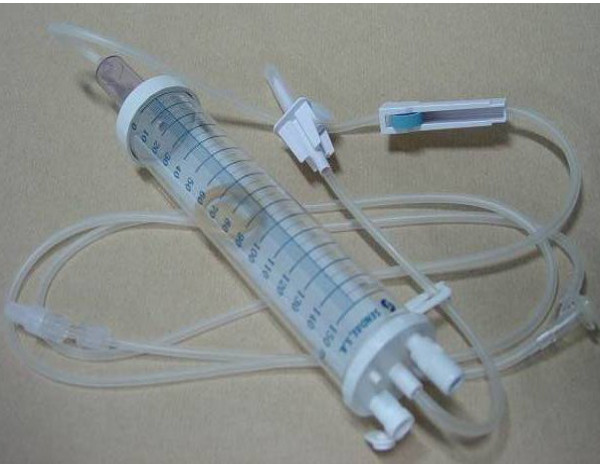
**Open Infusion Container - Burette with air filter**.

**Figure 4:**
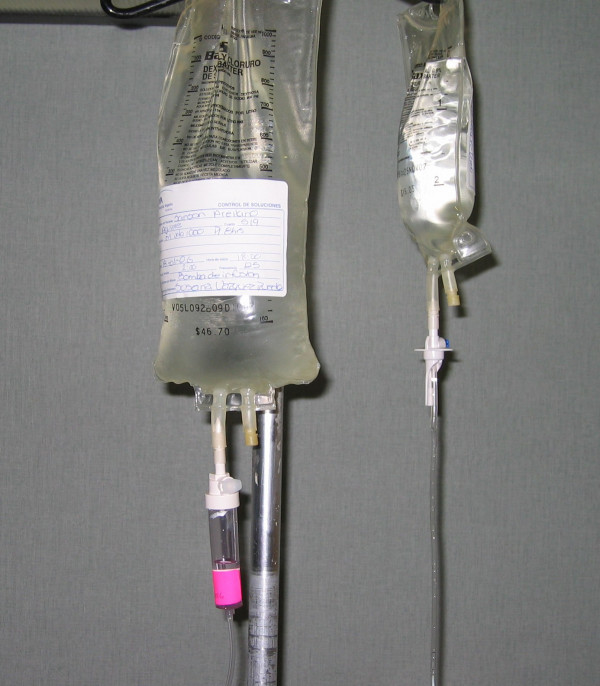
**Closed Infusion Container - Fully collapsible plastic container without air filter**.

Both open and closed infusion containers are used worldwide [5,17]. Open infusion containers consist of rigid (glass, burette) containers or semi-rigid plastic containers that must admit air (air filter or needle) to empty the contents (Figures 1, 2, 3). Closed infusion containers consist of fully collapsible plastic containers that do not require or use any external vent (air filter or needle) to empty the solution, and have self-sealing injection ports (Figure 4).

Standard practice incorporates the use of closed systems to prevent healthcare-associated infections (e.g., catheter-associated urinary tract infections [CAUTI]) [18], ventilator-associated pneumonia [VAP] [19], surgical site infections [20], and central line-associated-bloodstream infections [CLAB]) [3,5,17]. Numerous countries have reported outbreaks of infusion-related CLAB traced to contamination of infusate in open infusion systems [15,21-26]. Extrinsic or in-use contamination plays the most important role in bacterial contamination of the infusion system [26-28].

In Argentina, switching from open, semi-rigid plastic infusion containers to closed, fully collapsible plastic infusion containers was cost effective and resulted in a 64% CLAB rate reduction [17]. However, the open containers studied were limited to semi-rigid plastic containers, and time to CLAB was not studied in Argentina.

In this study conducted in Mexico, 3 types of open infusion containers (glass bottles, burettes, and semi-rigid plastic) were utilized. A time-to-onset of CLAB analysis was also included to evaluate the probability of developing CLAB.

We report the results of a prospective, sequential study undertaken to determine the impact of switching from an open (glass bottles, burettes, and semi-rigid plastic) to a closed, fully collapsible plastic infusion container (Viaflex^®^) on the rate and time-to-onset of CLAB in Mexico.

## Methods

### Setting

Three hospitals in Mexico City participated in the study: General Hospital, Century XXI Specialties Instituto Mexicano del Seguro Social (IMSS) Hospital, and Mancera IMSS Hospital. In accordance with recommendations [29], each has an active infection control program, composed of a physician trained in internal medicine and infectious diseases, and an infection control nurse. General Hospital is a public hospital, while Century XXI Specialties IMSS Hospital and Mancera IMSS Hospital are social security hospitals. The four intensive care units (ICUs) in the study centers operate at the highest level of complexity in Mexico (Level IV), providing treatment for patients who have undergone open-heart surgery, neurosurgery, gastrointestinal or orthopedic surgery, as well as patients with trauma or serious medical illnesses.

The study was conducted in accordance with the Declaration of Helsinki. The study protocol was approved by the ethics committee at General Hospital and Century XXI Specialties IMSS Hospital. Approval was not required by Mancera IMSS Hospital, as study specific procedures did not exceed the scope of standard medical care.

A subject informed consent letter, which detailed information regarding adverse effects, was approved by the ethics committee at General Hospital. However, subject informed consent was not required at either Century XXI Specialties IMSS Hospital or Mancera IMSS Hospital, as the study did not disclose the patient's confidentiality or privacy and did not involve any additional risks beyond the usual medical interventions performed in the participating ICUs.

### Data Collection

Patients who had a CL in place for >24 hours were enrolled from each of the study ICUs. A trained nurse prospectively recorded on case report forms the patient's gender, average severity-of-illness score (ASIS) on ICU entry [30], device utilization, antibiotic exposure, and all active infections identified while in the ICU. The decision to obtain blood cultures was made independently by the patient's physicians. Standard laboratory methods were used to identify microorganisms recovered from positive blood cultures [31].

### Definitions

United States Centers for Disease Control National Nosocomial Infections Surveillance Systems (CDC-NNIS) program definitions were used to define device-associated infections: CLAB (both laboratory-confirmed infection and clinical primary nosocomial sepsis), catheter-associated urinary tract infection CAUTI, and ventilator-associated pneumonia VAP [32].

An open infusion container was defined as a rigid (glass, burette) or semi-rigid plastic container that must admit air to empty (air filter or needle). A closed infusion container was defined as a fully collapsible, plastic container that does not require or use any external vent (air filter or needle) to empty the solution, and has injection ports that are self-sealing.

### Study Design

Active surveillance for CLAB and compliance with infection control practices continued throughout the study using CDC-NNIS methodologies, definitions, and criteria [30]. The open container period lasted 6 months (December 2002 to May 2003) at General Hospital, 5 months (February 2003 to June 2003) at Century XXI Specialties IMSS Hospital, and 4 months (April 2003 to July 2003) at Mancera IMSS Hospital. The closed container period lasted 6 months (June 2003 to November 2003), 5 months (July 2003 to November 2003), and 4 months (August 2003 to November 2003), respectively.

Baxter Viaflex^®^, a fully collapsible plastic closed infusion container, was used during the closed container period. Commercially available open infusion containers (glass container, semi-rigid plastic container, and burette products) were used during the open container period.

Protocol specified target hand hygiene (HH) and CL care compliance was set at ≥70% and ≥ 95%, respectively. We assessed HH (which included the use of alcohol based hand rub at one hospital) compliance [33], placement of gauze on CL insertion sites, condition of gauze dressing (absence of blood, moisture, and gross soilage; occlusive coverage of insertion site) [34,35], and documentation for date of CL insertion. A research nurse observed healthcare workers (physicians, nurses, and paramedical staff) three times per week across all work shifts and recorded information on a standard form. In addition, we conducted active surveillance for other common healthcare-acquired infections such as VAP and CAUTI. Mortality data also were collected.

### Data Analysis

**Figure 5:**
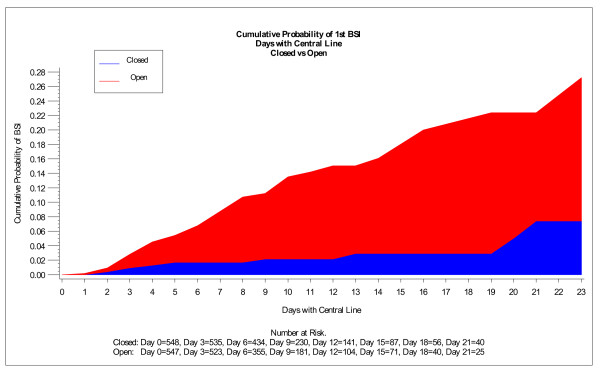
**Cumulative probability of 1^st ^CLAB displayed by CL days**.

Outcomes measured during the open and closed container periods included the incidence density rate of CLAB (number of cases/1000 CL days) and time to CLAB. Chi-square analyses for dichotomous variables and t-test for continuous variables were used to analyze baseline differences between periods. Relative risk (RR) ratios, 95% confidence intervals (CIs), and P-values were determined for all outcomes. Time to first CLAB was analyzed using a log-rank test and is presented graphically using Kaplan-Meier curves. In addition, simple life table conditional probabilities are presented graphically to help explain the changing risk of infection over time (Figure 5).

## Results

During the study, 1096 patients were enrolled, 548 during the open container period and 548 during the closed container period. Patients in both periods were statistically similar regarding patient demographics, underlying illness (except for endocrine diseases, cardiac failure, angina pectoris, chronic obstructive pulmonary disease, hepatic failure, and presence of previous infection), length of stay, ASIS, device utilization, and antibiotic usage (Table 1).

**Table 1:** Patient demographics, underlying illness, length of stay, device utilization and antibiotic usage during the two study periods

	Open infusion container N = 548	Closed infusion container N = 548	RR	95% CI	P-value
	**% (n/N)**	**% (n/N)**			
				
Sex (Male)	43.8% (240/548)	48.0% (263/548)	1.10	0.96 - 1.25	0.16
Sex (Female)	56.2% (308/548)	52.0% (285/548)	0.93	0.83 - 1.03	-
Endocrine Disease	21.0% (45/214)	30.3% (154/508)	1.44	1.08 - 1.93	0.01
Cardiac Failure	27.1% (58/214)	42.5% (216/508)	1.57	1.23 - 2.00	<0.01
Angina Pectoris	2.8% (6/214)	10.0% (51/508)	3.58	1.56 - 8.22	<0.01
Cardiac Surgery	0.0% (0/214)	0.4% (2/508)	-	-	0.36
COPD	0.0% (0/214)	15.0% (76/508)	-	-	<0.01
Cancer	4.2% (23/548)	4.2% (23/548)	1.00	0.57 - 1.76	1.0
Renal Impairment	4.7% (10/214)	7.5% (38/508)	1.60	0.81 - 3.15	0.17
Hepatic Failure	0.9% (2/214)	3.9% (20/508)	4.21	0.99 - 17.86	0.03
Abdominal Surgery	23.4% (128/548)	22.1% (121/548)	0.95	0.76 - 1.18	0.61
Thoracic Surgery	0.0% (0/214)	0.8% (4/508)	-	-	0.19
Trauma	0.0% (0/214)	1.2% (6/508)	-	-	0.11
Previous Infection	0.0% (0/214)	21.5% (109/508)	-	-	<0.01
Stroke	0.0% (0/214)	1.0% (5/508)	-	-	0.15
Immunodeficiency	0.9% (2/214)	1.6% (8/507)	1.69	0.36 - 7.89	0.50
Urinary catheter	91.2% (500/548)	94.0% (515/548)	1.03	1.00 - 1.07	0.08
Mechanical ventilator	66.4% (364/548)	68.2% (374/548)	1.03	0.95 - 1.12	0.52
	**Mean ± SD**	**Mean ± SD**			
				
ICU stay (days)	6.5 ± 6.56	7.1 ± 6.69	-	-	0.13
Age (yrs)	54.1 ± 18.29	54.4 ± 18.58	-	-	0.78
Severity-of-illness score	3.7 ± 0.90	3.8 ± 0.82	-	-	0.45
CL utilization per patient (days)	6.7 + 7.35	7.4 + 7.54	-	-	0.11
Mechanical ventilator utilization per patient (days)	4.3 ± 6.10	4.7 ± 6.61	-	-	0.24
Urinary catheter utilization per patient (days)	6.0 ± 6.64	6.6 ± 6.65	-	-	0.18
	**Defined daily dose**	**Defined daily dose**			
				
Antibiotic use per 1000 days	1485	1540	-	-	0.056

n, number of subjects with the characteristic present at baseline; N, total number of subjects with the with available (non-missing) results for the characteristic at baseline.

No new infection control interventions, training programs, products or technologies were introduced during the study periods and all of the investigators, key study personnel, classifications, and diagnostics techniques remained constant throughout the entire study. A lead-in period of at least 3 months duration was conducted to standardize HH and CL care compliance practice.

Compliance with HH during both periods was above 70% (81.0% and 74.7% during the open and closed container periods, respectively; RR = 0.92; 95% CI = 0.90 - 0.95).

Presence of gauze at CL site was 99.2% and 99.0% during the open and closed container periods, respectively (RR = 1.00; 95% CI = 0.99 - 1.00). Correct condition of gauze was 98.3% and 97.9% during the open and closed container periods, respectively (RR = 1.00; 95% CI = 0.99 - 1.00). Presence of the date at the CL insertion site/administration set was 100% during both the open and closed container periods.

The incidence density rate and percentage of patients with CLAB were both significantly lower in the closed container period compared to the open container period (Table 2). The majority of laboratory confirmed CLAB isolates in both the open (53.3%, 16/30) and closed (66.6%, 4/6) infusion container period were Gram-positive; Gram-negative isolates represented 43.3% (13/30) in the open container period and 33.3% (2/6) in the closed container period. The distribution of microorganisms for both container periods is shown on Table 3.

**Table 2:** Incidence of CLAB, CAUTI, VAP, and mortality during the two study periods

	Open infusion container N = 548	Closed infusion container N = 548	RR	95% CI	P-value
CL days no.	3661	4055	-	-	-
CLAB no.	59	13	-	-	-
CLAB per 1000 CL days	16.1	3.2	0.20	0.11 - 0.36	<0.01
Percentage of patients with CLAB	10.8	2.4	0.22	0.12 - 0.40	<0.01
Urinary catheter days no.	3302	3590	-	-	-
CAUTI no.	30	36	-	-	-
CAUTI per 1000 catheter days	9.1	10.0	1.10	0.68 - 1.79	0.69
Mechanical ventilator days no.	2344	2592	-	-	-
VAP no.	66	71	-	-	-
VAP per 1000 mechanical ventilator days	28.2	27.4	0.97	0.70 - 1.35	0.87
Deaths no.	128	88	-	-	-
Percentage of patients who died	23.4	16.1	0.69	0.54 - 0.88	<0.01

no., number; CL, central line; CLAB, central line-associated bloodstream infection; CAUTI, catheter-associated urinary tract infection; VAP, ventilator-associated pneumonia

**Table 3:** Microbial profile of CLAB during the two study periods

Microorganism	Open infusion container	Closed infusion container
CULTURE DOCUMENTED BSIs	30	6
Gram-positive bacteria, n (%)	16 (53.3%)	4 (66.6%)
*Staphylococcus aureus*	6	1
Coagulase-negative staphylococci	9	3
Enterococci species	1	0
Gram-negative bacteria, n (%)	13 (43.3%)	2 (33.3%)
Alcaligenes species	1	0
Enterobacter species	5	0
Klebsiella species	1	1
Proteus species	1	0
Acinetobacter species	2	0
Serratia species	1	1
Pseudomonas species	2	0
Yeasts, n (%)	1 (3.3%)	0
Candida species	1	0

In this study, we examined the timing of when the first CLAB was acquired comparing the open and closed container periods (Figure 5). The majority (62%) of patients had a CL in place for ≤4 days. When examining three-day intervals, the conditional probability of acquiring a CLAB in the closed container period was observed to be relatively constant (1.4% at Days 2-4 to 0.5% at Days 8-10). In the open container period, the conditional probability of acquiring a CLAB was higher in each three-day interval compared to the corresponding three-day interval in the closed container period. The conditional probability of acquiring a CLAB in the open container period ranged from 4.9% at Days 2-4 to 5.4% at Days 8-10.

Overall, the chance of a patient acquiring a CLAB decreased significantly-by 81%- in the closed container period (Cox proportional hazard ratio 0.19, P < 0.0001).

There was no statistically significant difference between the two periods with respect to incidence of CAUTI or VAP rate (Table 2).

Mortality during the closed container period (16.1%) was statistically significantly lower than during the open container period (23.4%) (RR = 0.69; 95% CI = 0.54 - 0.88; P = 0.002) (Table 2).

## Discussion

Critically ill patients commonly require CL access for administration of large volumes of IV fluid, medications, blood products, or for hemodynamic monitoring. All of these carry a greater risk of CLAB [4,5,10,11].

Studies have shown that CLABs increase length of stay, cost, and attributable mortality [2,3,5,36]. In 2005, Stone conducted a meta-analysis of all the cost studies published over a five-year period and found that the average cost of one CLAB was $36,441 (US) [36]. Previously, Rosenthal et al in Argentina published that CLAB resulted in an extra 12 days of hospitalization and $4,888 (US) [3]. Similarly, Higuera et al reported that CLAB in Mexico resulted in an extra six days of hospitalization and $11,560 (US) [2].

Moreover, CLABs are apparently related to increased attributable mortality: In an Australian study, CLABs resulted in excess mortality of 12% [37], and in a study from the United States, Pittet et al reported an attributable mortality of 25% [38,39]. Rosenthal et al likewise published an attributable mortality of 25% in a study of CLABs in medical/surgical ICUs in Argentina [12], and Higuera et al recently reported an attributable mortality of 20% in a study of ICUs in Mexico [2].

CLAB can be easily prevented [40]. The efficacy of simple interventions has been repeatedly documented in randomized trials. These interventions include, but are not limited to, mandating use of maximal barrier [41,42], HH, skin antisepsis, catheter site dressing regimens, catheter securement devices, and implementation of outcome and process surveillance plus education and performance feedback [34,35].

Contamination of infusate or catheter hubs have resulted in epidemics of infusion-related CLAB [43]. It is very rare in the United States to have intrinsic contamination of parenteral fluids (microorganisms introduced during manufacture) [43]. In addition, the risk of extrinsic contamination of infusate during administration in the hospital has been reduced with widespread use of closed infusion systems. However, open infusion systems are still widely used throughout the world. A high rate of CLAB was associated with use of open infusion containers in this study, whereas there was a significant reduction in CLAB rate with use of a closed infusion container. However, to date no regulations in Mexico require the use of closed infusion systems.

Open infusion containers may increase the risk of contamination and administration-related CLAB because they allow the entry of air into the system, thereby also providing an opportunity for microbial entry. The closed infusion container that was investigated (a fully collapsible, plastic container not requiring or using any external vent [air or needle] to empty and having self-sealing ports) was designed to overcome this flaw. When infusing intravenous solutions, it is important that the residual solution in the container after the infusion not exceed 5% of the nominal volume in order to deliver the correct dosage. Containers leaving more than this amount, even if non-vented and theoretically closed, may nevertheless be vented in practice by healthcare workers who spike the container with a needle to ensure delivery of the solution to the patient, thus introducing an opportunity for contamination. Similarly, when choosing a closed system for parenteral infusion, it is important that the container enables a consistent and even infusion rate throughout the administration process without the assistance of a mechanical device like an infusion pump, and that it maintains its integrity during extreme usage conditions.

The probability of developing a CLAB was assessed in three-day intervals during each period to evaluate the effect of CLAB over time. It is useful to display and assess the distribution of time of CL use across patients in order to avoid being misled by a cross study comparison when comparing results of CLABs between studies. A study with a preponderance of 2-4 CL days per patient compared to a study with a preponderance of 10-12 CL days per patient would have vastly different observed CLAB rates when a hazard function is not constant - even when all other factors are identical (e.g., equal number of total CL days, similar patient population, and identical study design and methodology).

We also examined the timing of when the first CLAB was acquired comparing open versus closed infusion containers. Prior investigation published in 2004 did not include this additional analysis [17]. From this additional analysis, we demonstrated that when using an open infusion container, the risk of acquiring CLAB increases over time. However, if the patient receives infusate via a closed infusion container, the probability of acquiring a CLAB remains relatively constant. The patient also acquires a CLAB significantly later, suggesting that closed infusion containers reduce risk of CLAB acquisition over time. Subsequently, the use of a closed infusion containers could especially benefit those patients with more severe illness who may require CLs for longer periods of time. The delayed onset of CLAB may also benefit patients with CLs early during the course of treatment when their underlying illness might be most severe.

Blinding the treatment assignment was not practical in this study; however, this might have avoided the possible selection bias that could occur in an open label, randomized study. In an effort to minimize protocol violations, concurrent randomization of patients to the two infusion systems was also not implemented. Logistically it was easier to assure that patients received IV fluids in the appropriate infusion containers when only those containers were present in the ICUs during the specified open or closed container period of the study. Subsequently, a time series, sequential design was implemented. In order to minimize the effects of confounding factors no new infection control interventions, training programs, products or technologies were introduced during the study periods and all of the investigators, key study personnel, classifications and diagnostic techniques remained constant throughout the study. In addition, HH and CL care compliance practices were standardized during the lead-in period.

Another study limitation was that the study design did not allow for determination of the epidemiologic mechanisms responsible for the striking differences in outcome (e.g., reduced contamination of infusate). In spite of a reduction in HH compliance during the closed infusion system phase, the CLAB rate improved.

In a separate study by Munoz et al (1997), infusions were cultured at a second-level general teaching hospital in Mexico wherein a 29.6% contamination rate was found during the baseline period [23]. A 2% contamination rate was reported by Macias et al (1999) in a multi-center cross-sectional study in Mexico; lapses in aseptic technique, and breaks in the infusion system while injecting IV medications were risk factors for in-use contamination [15].

The CDC Healthcare Infection Control Practices Advisory Committee (HICPAC) offers direction for the prevention of CLAB by limiting manipulations of and entry into running infusions. In addition, persons handling or entering an infusion should only do so after implementing appropriate infection control measures and with strict adherence to aseptic technique [42].

## Conclusions

This study has demonstrated that the use of closed infusion containers prevents CLAB and reduces mortality. Hospitals that continue to use burettes and/or open rigid or semi-rigid fluid containers (which must be vented to allow ambient air entry and fluid egress) should evaluate switching to closed, non-vented, fully collapsible bags to reduce CLAB rates.

## Competing interests

VDR serves as a consultant to Baxter. Baxter is providing the article-processing charge for this manuscript.

## Authors' contributions

VDR provided the study concept and design, served as the study coordinator, analyzed the data, and drafted/revised the study manuscript. MSRF, FHR, and JMS each acquired study data as principal investigators at their respective hospitals, and provided critical review of the content of the manuscript. All authors read and approved the final manuscript.
